# Exomap1 mouse: A transgenic model for *in vivo* studies of exosome biology

**DOI:** 10.1016/j.vesic.2023.100030

**Published:** 2023-10-10

**Authors:** Francis K. Fordjour, Sarah Abuelreich, Xiaoman Hong, Emeli Chatterjee, Valeria Lallai, Martin Ng, Andras Saftics, Fengyan Deng, Natacha Carnel-Amar, Hiroaki Wakimoto, Kazuhide Shimizu, Malia Bautista, Tuan Anh Phu, Ngan K. Vu, Paige C. Geiger, Robert L. Raffai, Christie D. Fowler, Saumya Das, Lane K. Christenson, Tijana Jovanovic-Talisman, Stephen J. Gould

**Affiliations:** a Department of Biological Chemistry, Johns Hopkins University, Baltimore, MD, 21205, USA; b Department of Cancer Biology and Molecular Medicine, Beckman Research Institute, City of Hope, Duarte, CA, 91010, USA; c Department of Cell Biology and Physiology, University of Kansas Medical Center, Kansas City, KS, 66160, USA; d Cardiovascular Research Center, Massachusetts General Hospital, Harvard Medical School, Boston, MA 02114, USA; e Department of Neurobiology & Behavior, University of California Irvine, Irvine, CA, 92697, USA; f Northern California Institute for Research and Education, San Francisco, CA, 94121, USA; g Department of Neurosurgery, Massachusetts General Hospital, Harvard Medical School, Boston, MA, 02114, USA; h Department of Veterans Affairs, Surgical Service (112G), San Francisco VA Medical Center, San Francisco, CA, 94121, USA; i Department of Surgery, Division of Vascular and Endovascular Surgery, University of California, San Francisco, CA, 94143, USA

**Keywords:** CD81, Cre recombinase, Extracellular vesicle, EV, Exosome biogenesis, Exosome reporter

## Abstract

Exosomes are small extracellular vesicles (sEVs) of ~30–150 nm in diameter that are enriched in exosome marker proteins and play important roles in health and disease. To address large unanswered questions regarding exosome biology *in vivo*, we created the Exomap1 transgenic mouse, which in response to Cre recombinase expresses the most highly enriched exosomal marker protein known, human CD81, fused to mNeonGreen (HsCD81mNG), and prior to Cre expresses a mitochondrial red fluorescent protein. Validation of the *exomap1* mouse with eight distinct Cre drivers demonstrated that HsCD81mNG was expressed only in response to Cre, that murine cells correctly localized HsCD81mNG to the plasma membrane, and that this led to the secretion of HsCD81mNG in EVs that had the size (~70–80 nm), topology, and composition of exosomes. Furthermore, cell type-specific activation of the *exomap1* transgene allowed us to use quantitative single molecule localization microscopy to calculate the cell type-specific contribution to biofluid exosome populations. Specifically, we show that neurons contribute ~1% to plasma and cerebrospinal fluid exosome populations whereas hepatocytes contribute ~15% to plasma exosome populations, numbers that reflect the known vascular permeabilities of brain and liver. These observations validate the use of Exomap1 mouse models for *in vivo* studies of exosome biology.

## 1. Introduction

Mammalian cells secrete two broad classes of extracellular vesicles (EVs), the exosomes and other small EVs (sEVs) that are <200 nm in diameter, and the microvesicles and other large EVs (lEVs) that are>200 nm in diameter.^1–7^ Of these, the exosomes are the most intriguing,^1,3,7^ as they are highly enriched in specific subsets of exosome cargo proteins, especially the exosomal tetraspanins CD81, CD9, and CD63 (5,8,9), and their associated scaffold proteins (e.g. syntenin^8,9^. In contrast, the lEVs (i.e., microvesicles, platelets, apoptotic bodies, oncosomes, etc.) do not appear to be enriched in any particular set of proteins or RNAs and have a molecular composition similar to the cell.^1,7^

It is well established that exosomes transmit signals, molecules, and nutrients to other cells, both nearby and far away,^10–23^ while also modifying the extracellular environment (matrices, interstitial and other biofluids^24–27^) and facilitating protein quality control in exosome-producing cells.^28–31^ Not surprisingly, exosomes have been implicated in numerous physiological and disease processes, are abundant in all biofluids, and hold high potential as carriers of vaccines and therapeutics.^1,3^ However, most of what we know about exosome biogenesis, composition, and function has come from *in vitro* studies. This has left a gap in knowledge about how exosomes are made by different cell types *in vivo*; the extent to which different cell types contribute to the exosomes found in blood, lymph, and other biofluids; the amount and specificity of exosome traffic between different cell types, tissues, organs and organ systems; and how these processes change in response to different states of health and disease.

Of the known exosome marker proteins, CD81 is 15-fold more enriched in exosomes than CD63 and 3-fold more enriched than CD9^5,6,32^. Furthermore, CD81 shows no enrichment in the larger, microvesicle class of EVs that are >200 nm making it the most specific marker of exosomes yet described^32^ (though like all plasma membrane proteins it can occasionally be found in lEVs). In addition, CD81 is a small (~25 kDa) polytopic integral membrane protein to which species-specific monoclonal antibodies are available and bind to its extracellular domain. These properties make human CD81 an ideal marker for *in vivo* studies of exosome biogenesis in a murine system. We therefore designed a transgenic mouse to carry the *exomap1* transgene, which expresses a mitochondrial form of TdTomato from the CAG promoter, and in response to Cre recombinase expresses human CD81 (HsCD81) fused to the bright mNeonGreen^33^ (mNG) protein (HsCD81mNG). We also demonstrate that HsCD81mNG is properly localized to the plasma membrane, is properly loaded into exosome-sized vesicles that have the size, topology, and composition of exosomes, and that tissue-specific expression of HsCD81mNG can be used to measure the tissue-specific contributions to murine biofluid populations.

## 2. Methods

### 2.1. Nucleic acids

The plasmid pFF077 was assembled by ligating synthetic DNAs carrying the Amp gene, bacterial origin of replication, attB1 segment,^34^ the CAG enhancer/promoter,^35^ a codon-optimized ORF that encodes MTS-tdTomato^36^ flanked by two loxP511 sites,^37^ the HsCD81mNG ORF from pJM1358 (37), the WPRE element,^38^ the bGH polyadenylation signal^39^ and the attB2 segment.^34^ The MTS-tdTomato ORF was codon-optimized to minimize recombination between the direct DNA repeats present in the original tdTomato ORF(35). The plasmid pJM776 expresses iCre, a codon optimized form of Cre recombinase.^40^ The mRNA encoding ΦC31 integrase was synthesized as previously described.^34^

### 2.2. Cell culture and transfection

HEK293 cells were obtained from ATCC (CRL-1573) and grown in complete media (CM; DMEM, 10% fetal bovine serum, 1% penicillin/streptomycin). HEK293 cells were transfected with lipofectamine 3000 according to the manufacturer’s instructions (Thermo).

### 2.3. Fluorescence microscopy of HEK293 cells

HEK293 cells transfected with either pFF077 or co-transfected with pFF077 and pJM776 were seeded onto sterile, poly-d-lysine-coated cover glasses, grown overnight, then fixed (3.7% formaldehyde in PBS for 30 min), permeabilized (1% Triton X-100 in PBS for 5 min), stained with DAPI, washed, and then mounted on glass slides. The cells were examined using BH2-RFCA microscope (Olympus) equipped with an Olympus S-plan Apo 63 × 0.40 oil objective and a Sensicam QE (Cooke) digital camera using IPLab 3.6.3 software (Scanalytics, Inc.). Images were processed in Adobe Photoshop and figures were assembled in Adobe Illustrator.

### 2.4. Preparation of sEV/exosomes from tissue culture supernatants

The analysis of exosomes released by HEK293 cells was carried out by culturing transfected HEK293 cells for three days, after which the conditioned media was collected and the cells were lysed in SDS-PAGE sample buffer. Exosomes were purified from conditioned media as previously described,^5,41^ with contaminating cells and cell debris removed by centrifugation at 5000×*g* for 5 min, followed by filtration through a 200 nm pore diameter size sterile filtration unit. The resulting clarified tissue culture supernatant was then spun at 10,000×*g* for 30 min, twice, to ensure removal of any residual large entities, and then spun at 100, 000×*g* for 2 h to pellet sEVs. Exosome pellets were then resuspended in SDS-PAGE sample buffer, and cell and sEV lysates were stored at −80 °C for later analysis.

### 2.5. Immunoblots

Protein samples were generated by boiling cell and exosome samples in SDS-PAGE sample buffer. Mouse tissue proteins were generated by harvesting tissues of interest (liver, kidney, brain), mincing with a razor blade in PBS in a sterile tissue culture plate. Cells were then pelleted in a 2 mL microfuge tube and resuspended in 200 μL RIPA buffer (ThermoFisher Scientific, Cat. No. 89900, Waltham, MA, USA) and 2 μL of Phosphatase/Protease inhibitor cocktail (ThermoFisher Scientific 78,440). One stainless steel bead (5 mm) was added to each tube, the tubes were inserted into a TissueLyser (Qiagen), and agitated at 50 vibrations/min until the tissue was homogenized properly. Insoluble material and beads were removed by low-speed centrifugation for 1 min, and the resulting supernatant was clarified by centrifugation at 12,000×*g* for 10 min at 4 °C. The resulting cell lysate supernatants were transferred to new tubes and stored −80 °C.

Protein samples in 1x SDS-PAGE sample buffer were separated by SDS-PAGE and electrophoretically transferred to PVDF membranes. Membranes were blocked, incubated in blocking solution containing primary antibody overnight at 4 °C, washed, and incubated with an HRP conjugate of secondary antibody for 2 h, then washed again. Membranes were incubated with ECL detection reagent and imaged using a digital light capture device. Gel images processed in Adobe Photoshop and figures were assembled in Adobe Illustrator. Unlabeled and labeled antibodies specific for human CD81, mouse CD9, mouse CD81, as well as control rat, hamster, and human IgG were acquired from commercial sources (Biolegend).

### 2.6. Mice, gDNA extraction, and PCR

All procedures were conducted in accordance with the NIH Guide for the Care and Use of Laboratory Animals and were approved by the Institutional Animal Care and Use Committees of each participating laboratory. Mice were maintained in environmentally controlled vivariums, with food and water provided *ad libitum*. Adult mice were generated from breeding colonies at the Johns Hopkins University (*exomap1* founder line), Charles River laboratories (*exomap1* stock line), Massachusetts General Hospital, University of Kansas Medical Center, University of California Irvine, and University of California San Francisco/Veterans Administration, San Francisco.

Pronuclear injection of H11P3 zygotes was with linearized pFF077 DNA and ΦC31 integrase mRNA. Injected zygotes were implanted into pseudo-pregnant females as previously described (the H11P3 mouse has three copies of attP in the mouse Hipp11 (H11) locus^34^). Pups were assessed for transgene integration by PCR analysis of tail snip genomic DNA using primers specific for the transgene and for sequences specific for the engineered H11P3 locus.^34^ Primers flanking the three attP sites in the H11P3 locus and internal to the *exomap1* transgene revealed that the MT124 founder mouse carried a single insertion of the *exomap1* transgene between attP sites 2 and 3 of the H11P3 locus. The MT124 mouse line was established by backcrossing the original *exomap1*^+/^ founder mouse to C57Bl/6 mice six times, with transgene carrier status monitored by fluorescence imaging of mice or mouse tail snips, and by PCR analysis of mouse tail snip gDNA using primers flanking the 5′ transgene insertion junction (5′-GGTGATAGGTGGCAAGTGGTATTCCGTAAG-3′ and 5′-CATA-TATGGGCTATGAACTAATGACCCCGT-3′, which yields a 447 bp-long DNA fragment) and/or the 3′ transgene insertion junction (5′-GCATCGCATTGTCTGAGTAGGTGTCA-3′) and 5′-CCGCGAAGTTCCTATA CCTTTTG-3′, which yields a 301 bp-long DNA fragment). The extraction of gDNA from mouse tail snips and their analysis by PCR was performed using standard procedures.

Cre driver mice used in this study included the *Zp3*-Cre line C57BL/6-Tg (Zp3-cre)93Knw/J (Jackson Laboratories stock #003651 (42)), the *Aromatase*-Cre line *cyp19a1*-Cre (generous gift from Dr. Jan A. Gossen,^42^ the *LysM*-Cre line B6.129P2-Lyz2tm1 (cre)Ifo/J (Jackson Laboratories stock #004781 (44)), the *HSA*-MCM line Tg (ACTA1-cre/Esr1*)2Kesr/J (Jackson Laboratories stock #025750 (45)), the *Dat*-Cre line Slc6a3tm1 (cre)Xz/J (Jackson Laboratories stock #020080 (46)), and the *Camk2a*-Cre line B6. Cg-Tg (Camk2a-cre)T29–1Stl/J (Jackson Laboratories stock #005359 (47)). Double transgenic F1 mice were obtained by crossing hemizygous Cre driver lines with hemizygous *exomap1*^+*/*^ or homozygous *exomap1*^+*/*+^ mice, with the presence of the *exomap1* transgene determined by fluorescence illumination of mice, or by extraction of genomic DNA from mouse tail snips and PCR analysis. The presence of Cre driver transgenes was determined by gDNA PCR analysis.

### 2.7. Virus infections

The AAV5/*Rpe65*-Cre virus was obtained from Vigene Biosciences (pAAV-Rpe65-Cre, AAV5 serotype, titer >1×10^13–10^14 gene copies/mL). Subjects were anesthetized with a 1–3% isoflurane/oxygen mixture and positioned in a Kopf stereotaxic frame with the incisor bar set to the flat-skull position. Brain microinjections were administered into the dorsal third ventricle at a volume of 2 μL at a rate of 0.1 μL per minute across 20 min, and the injector remained in place for an additional 5 min to allow for diffusion. The coordinates were as follows: AP −1.00 mm, ML: midline at bregma, DV −2.07 mm. The mice were sacrificed at least 2.5 after injection. Mice were then perfused with 4% paraformaldehyde, and organs were removed and placed in 30% sucrose. At least 72 h thereafter, tissue was sectioned on a cryostat, mounted onto microscope slides, coverslipped with Vectashield mounting media containing DAPI, and then examined by fluorescence microscopy. n = 3 subjects were examined to verify expression patterns.

The AAV8/*TBG*-Cre viruses were obtained from commercial suppliers (1×10^13 genome copies (GC)/mL, CV17191-AV8, Charles River; or 2×10^13 GC/mL, 107,787-AAV8, Addgene), diluted 1:20 into a final volume of 100 μL PBS, and delivered by intravenous injection into either the tail vein or retro-orbital sinus. Animals were maintained for 2–4 weeks post-injection, after which tissue and blood samples were collected.

### 2.8. Fluorescence microscopy of mouse cells and tissue sections

For the analysis of oocytes and ovarian tissues, mature female *exomap1*:*Zp3*-Cre and *exomap1*:*Cyp19a1*-Cre mice were sacrificed and ovaries were removed. Follicles were mechanically ruptured to disperse oocytes and somatic follicle (granulosa/cumulus) cells, and the dispersed cells were examined by fluorescence microscopy.

For fluorescence microscopy of tissue sections, mice were euthanized, and then organs and tissues of interest removed and placed in 4% paraformaldehyde overnight, followed by 30% sucrose. 3 days later, tissues were sectioned on a cryostat at 35 mm thickness, directly mounted onto microscope slides, then coverslipped with Vectashield with DAPI (Vector Labs, Cat# H-1200) and examined by fluorescence microscopy.

### 2.9. Immunohistochemistry

Mice were anesthetized, perfused with 4% paraformaldehyde, and organs were removed and placed in 30% sucrose. Three days later the tissues were frozen, sectioned (4 μm) using a cryostat CM1850 (Leica, CA), mounted onto microscope slides, incubated with *anti*-HsCD81 monoclonal antibody linked to horseradish peroxidase, washed, and then processed for HRP immunohistochemistry.

### 2.10. Blood, plasma, serum, and CSF collection

Blood samples were isolated by either *retro*-orbitual bleeding using Micro-hematocrit capillary tubes (Fisher Scientific), submandibular bleeding, or cardiac puncture into EDTA. Plasma samples were generated from bloods by immediately spinning EDTA bloods (within 60 min of collection) at 2000×*g* for 10 min, followed by a second spin at 3000×*g* for 10 min. Plasma samples were stored at −80 °C. To isolate leukocytes, blood cells were collected by low-speed centrifugation, resuspended and incubated in ACK RBC lysis buffer according to the manufacturer’s instructions (Thermo), followed by pelleting of leukocytes by low-speed centrifugation and resuspension once again in ACK RBC lysis buffer. Leukocytes were then once again pelleted by low-speed centrifugation, after which they were interrogated by flow cytometry. Serum samples collected by cardiac puncture were allowed to clot for 20–30 min and then centrifuged at 3000×*g* for 10 min.

CSF samples were isolated from anesthetized mice as described.^43^ Briefly, the mouse’s head was fixed to a stereotaxic frame in the position that allowed the maximum opening of the cisterna magna. A Hamilton syringe (The Hamilton Company, Boston MA, USA) equipped with a 26-gauge needle or pulled glass pipette tip was inserted stereotactically in parallel to the brain axis to reach the cistern. The clear CSF (approximately 10 μL) was then drawn slowly to minimize possible contamination by blood or tissue.

### 2.11. Flow cytometry

Flow cytometry of circulating leukocytes was performed by first blocking Fc receptors by incubating cells with TruStain FcX PLUS (anti-mouse CD16/32, Biolegend) for 10 min. Cell were then incubated with anti-CD45 & anti-CD11b (Biolegend), followed by purification of CD45^+^, CD11b + cells on affinity capture beads. Fluorescence of MTS-tdTomato and HsCD81mNG in the CD45^+^, CD11b + cell population was determined by flow cytometry using a Beckman Coulter CytoFLEX S cytometer. Histograms of fluorescence intensity were processed in Adobe Photoshop and figures were assembled in Adobe Illustrator.

### 2.12. Isolation of exosomes/sEVs

Plasma and serum exosomes/sEVs were isolated by size exclusion column chromatography using pre-packed qEV columns (Izon), and also by immunoaffinity purification onto glass coverslips functionalized with anti-exosomal marker antibodies, and by immunoaffinity purification on SPIR imaging chips functionalized with anti-exosomal marker antibodies.

### 2.13. Nanoparticle tracking analysis (NTA)

*Exomap1*:*Dat*-Cre male and female mice were used to obtain samples of CSF (n = 15). CSF was first filtered with a 200 nm pore diameter cellulose acetate filter (Costar, RNase/DNase free, Cat#8161) and spun for 30 s. Samples were then diluted 1:20 with nanopure H_2_O. The size distribution pattern of CSF sEVs was quantified with a NanoSight NS300 and nanoparticle tracking analysis (NTA) software version 3.3 with sCMOS camera and either (***i***) no filter (brightfield) to examine all EVs in the sample, or (***ii***) a green light filter to detect EVs with HsCD81mNG fluorescence. The camera settings were as follows: level 6, slider shutter 86, slider gain 15, 25.0 frames per second, number of frames 1498, temperature 21.7 °C, syringe volume 0.6–1 mL, and syringe pump speed 100.

### 2.14. Quantitative single molecule localization and total internal reflection fluorescence microscopy

Details of single extracellular vesicle nanoscopy (SEVEN) protocol, which combines affinity isolation and quantitative single molecule localization microscopy (qSMLM), have been described previously.^44,45^ Briefly, 25 mm diameter coverslips #1.5H (Thermo Fischer Scientific, Cat# NC9560650; Waltham, MA, USA) were functionalized with N-hydroxysuccinimide (NHS) groups, followed by covalent attachment of monoclonal antibodies that bind to epitopes in the ectodomain of either the mouse CD81 or human CD81 proteins. Raw biofluid samples from one *exomap1*:*Camk2a*-Cre mouse, one *exomap1*^+/^ mouse infected with AAV8/*TBG*-Cre virus, and one control *exomap1*^+/^ mouse were diluted in PBS supplemented with 0.025% Tween 20 to a final volume of 50 μL and placed on the surface of these antibody-coated coverslips at room temperature overnight in a humidified chamber. For the CSF samples, 1 μL of CSF was used for incubation with the anti-mouse CD81-functionalized coverslip and 6 μL of CSF were used for incubation with the anti-human CD81-functionalized coverslip. For the plasma samples, 1 μL of plasma was used for incubation with the anti-mouse CD81-functionalized coverslip and 5 μL of plasma were used for incubation with the anti-human CD81-functionalized coverslip. These coverslips were then washed with PBS containing 0.025% Tween 20 and EVs were labeled with one of the following affinity reagents: (***i***) a mixture of AF647-labeled antibodies specific for mouse CD9 (Biolegend, Cat. No. 124802, San Diego, CA, USA), mouse CD63 (Biolegend, Cat. No. 143902, San Diego, CA, USA), mouse CD81 (Biolegend, Cat. No. 104902, San Diego, CA, USA), and human CD81 (Biolegend, Cat. No. 349502, San Diego, CA, USA) (*exomap1*:*Camk2a*-Cre mouse and control *exomap1*^+/^ mouse); (***ii***) a mixture of AF647-labeled antibodies specific for mouse CD9, mouse CD63, and mouse CD81 (*exomap1*^+/^ mouse infected with AAV8/*TBG*-Cre virus and control *exomap1*^+/^ mouse); (***iii***) AF647-labeled antibodies specific for human CD81 (*exomap1*^+/^ mouse infected with AAV8/*TBG*-Cre virus and control *exomap1*^+/^ mouse). All antibodies were fluorescently labeled as described previously at a molar ratio of ~1 (50). Samples were fixed and stored as described previously.^46,47^

For imaging, coverslips were placed in Attofluor cell chambers (ThermoFisher Scientific) loaded with direct stochastic optical reconstruction microscopy (dSTORM) imaging buffer.^48^ N-STORM super-resolution microscope (Nikon Instruments; Melville, NY, USA) was used for total internal reflection fluorescence (TIRF) and SMLM imaging using 488 nm and 640 nm lasers, respectively, using microscope components described previously.^49^ Images (10 regions of interest (ROI) for CSF samples and 20 ROI for plasma samples) were acquired using NIS-Elements software (Nikon Instruments). SMLM images were processed using N-STORM Offline Analysis Module of the NIS-Elements software to localize peaks. The data were analyzed with Matlab R2022a (MathWorks; Natick, MA, USA) using the Voronoi tessellation algorithm^46,47^ with a minimum of 40 localization points per cluster and an average of 14 localizations per single fluorescent tetraspanin antibody.

### 2.15. SPIR-IFM imaging

Custom SPIR imaging chips derivatized with antibodies specific for mouse CD81, mouse CD9, and human CD81, and with control IgG from rat, hamster, and human, were generated by Nanoview (Waltham, MA). Serum samples were collected from control mice, *exomap1*+/mice, and *exomap1*:AAV8/*TBG*-Cre mice (4 days post-infection) and sEVs were collected by filtration and SEC. sEVs/exosomes were then incubated on the SPIR imaging chips overnight at RT, washed, incubated with Alexa Fluor 555-labeled anti-human CD81, washed again, and interrogated by interferometric reflectance imaging to detect exosomes/sEVs, and by conventional fluorescence microscopy to measure vesicle-associated (***i***) HsCD81mNG fluorescence and (***ii***) Alexa Fluor 555 fluorescence.

## 3. Results

### 3.1. Design and validation of the exomap1 transgene

**Fig. 1.**
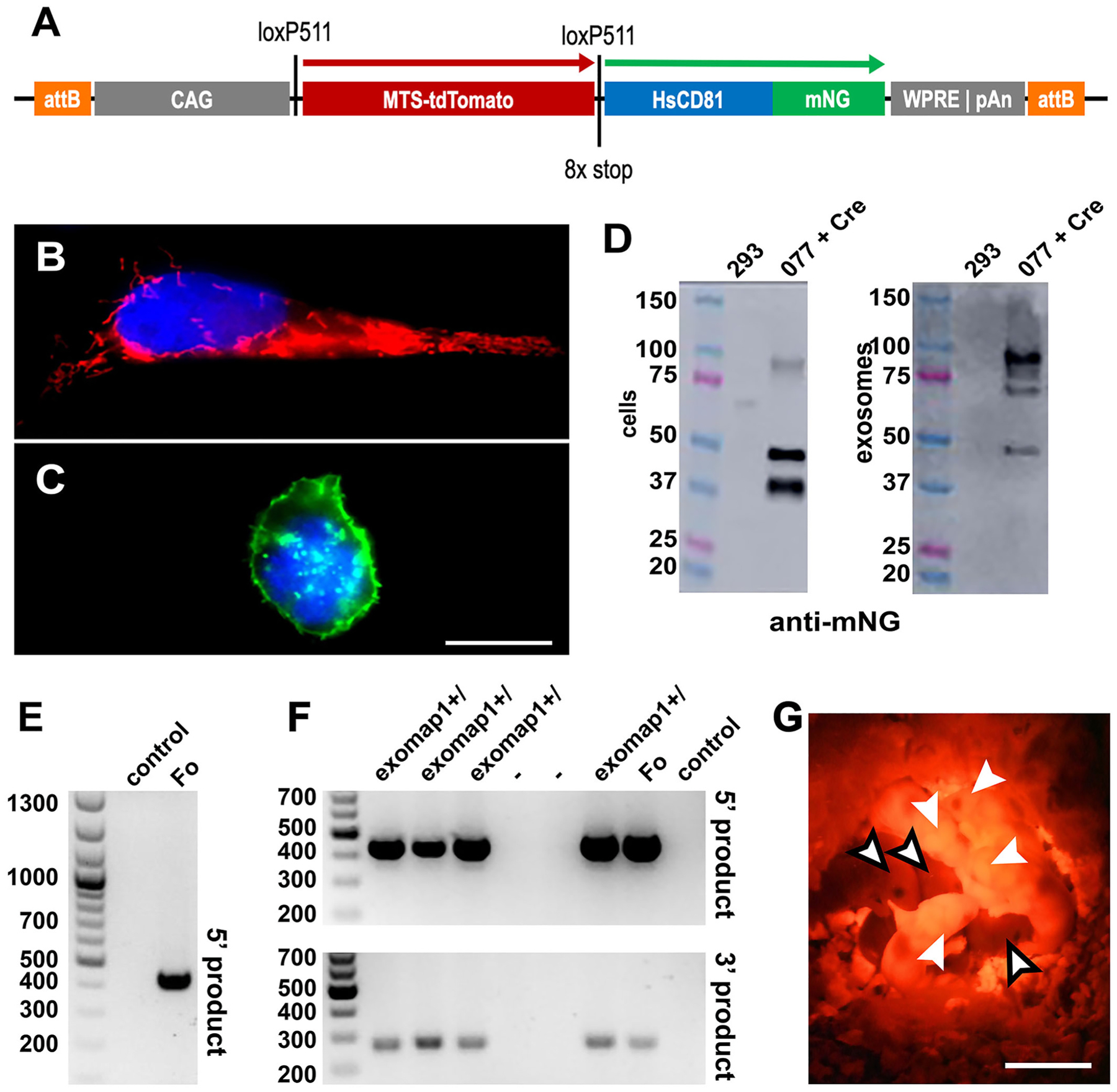
Design, genetics, and red fluorescence of exomap1+/− mice. (**A**) Line diagram of the *exomap1* transfer vector. (**B**, **C**) Fluorescence micrographs of DAPI-stained HEK293 cells transfected with (B) pFF077 or (C) pFF077 + pJM776 that had been fixed and stained with DAPI. Bar, 10 μm. (**D**) Anti-CD81 immunoblot of cell and exosome fractions of (left lanes) HEK293 cells and (right lanes) HEK293 cells expressing HsCD81mNG. MW size standards are in kDa. (**E**) Ethidium bromide-stained agarose gel electropherogram of genomic DNA (gDNA) PCR products generated using gDNAs extracted from (control) non-transgenic mouse and the (Fo) *exomap1*^+/^ founder mouse using the H11 locus 5′ primer and the CAG promoter 3′ primer, showing the 447 bp product that is diagnostic for the *exomap1* transgene. MW size markers are in bp. (**F**) Ethidium bromide-stained agarose gel electropherograms of PCR reaction carried out with gDNAs extracted from the tails of six F1 progeny from a cross between the (Fo) founder mouse and a non-transgenic control mouse, as well as gDNAs from the founder mouse and a non-transgenic control mouse. Upper panel shows products obtained using the H11 locus 5′primer and the CAG promoter 3′ primer. Lower panel shows products obtained using the bGH-pA 5′ primer and the FRTR2 3′ primer, with an expected *exomap1* transgene-specific product of 311 bp. MW size markers are in bp. (**G**) Fluorescence micrograph of pups from a cross of a control mouse with an *exomap1*^+/^, mouse, illuminated with green light and imaged with a red filtered camera. White arrows point to heads of four *exomap1*^+/^ carrier mice, readily identifiable by their red fluorescent ears, feet, and tails. Black bordered arrows point to the heads of three non-transgenic littermates. Bar, 1 cm.

The *exomap1* transgene plasmid pFF077 is a ΦC31 integrase donor^50^ that carries a Cre-regulated transgene flanked by attB sites (Fig. 1A), and is designed for future integration into the attP sites of the H11P3 mouse line (the H11P3 mouse line carries three attP sites integrated at the Hip11 safe harbor locus^34^). The *exomap1* transgene itself consists of the CAG promoter, a loxP5 site, an open reading frame (ORF) encoding the mitochondrial red fluorescent protein MTS-tdTomato, multiple stop codons in each reading frame, a second loxP site, the HsCD81mNG ORF^51^, and a 3’ untranslated region containing both the woodchuck hepatitis virus post-transcriptional regulatory element (WPRE)^38^ and the polyadenylation signal from the bovine growth hormone gene (bGH).

To ensure that the *exomap1* transgene in pFF077 expressed red fluorescence in the absence of Cre and green fluorescence in the presence of Cre, we transfected pFF077 into HEK293 cells on its own or together with a Cre recombinase-expressing plasmid (pJM775). The next day, the transfected cells were fixed, stained with DAPI, and examined by fluorescence microscopy. Cells transfected with pFF077 alone expressed bright red fluorescence in structures that had the expected morphology of mitochondria (Fig. 1B). In contrast, cells that had been co-transfected with pFF077 and pJM775 displayed bright green HsCD81mNG fluorescence, much of which was properly localized to the plasma membrane^5,6^ (Fig. 1C). It should be noted that we previously reported the development of HsCD81mNG and established its utility as an exosome marker protein^51^.

Immunoblot analyses (Fig. 1D) revealed that most of the exosomal HsCD81mNG migrated at ~100 kDa or slightly lower, with only a small amount of ~50 kDa monomer present. This was consistent with our prior discovery that oligomerization is a critical determinant of protein loading into exosomes.^52^ In contrast, the cell-associated forms of HsCD81 were primarily the ~50 kDa monomer and an ~40 kDa breakdown product.

### 3.2. Genetics of the Exomap1 mouse

To make the Exomap1 mouse, pFF077 was linearized outside the attB sites by restriction enzyme cleavage. This linear DNA donor fragment was purified, mixed with mRNA encoding the Φ31 integrase protein, and injected into the pronucleus of zygotes of the H11P3 mouse.^34^ Injected zygotes were implanted into pseudo-pregnant females and the resulting pups were screened by PCR of genomic DNA (gDNA). A single founder (MT124) was used for all subsequent studies. This mouse carried the *exomap1* transgene between attP sites #2 and #3 of the H11P3 locus. Presence of the *exomap1* transgene was tracked through horizontal transmission by genomic DNA extraction and PCR reactions using primers that flank the 5′ attP/B insertion site and the 3’ attB/P insertion site (Fig. 1E and F).

Hemizygous *exomap1*^+/^ mice expressed MTS-tdTomato throughout the body, allowing the visual identification of mice that carry the *exomap1* transgene, upon proper illumination and visualization (Fig. 1G). Husbandry of *exomap1*^+/^ mice revealed that the *exomap1* transgene was inherited in a Mendelian pattern. Within a set of 21 crosses of homozygous *exomap1*^+/+^ mice (for the 29 months of 08/2020 to 01/2023), the average litter size was 6, the sex ratio was 1, and no obvious adverse effects were associated with either hemizygous or homozygous carrier status. Of the 10 animals that were kept for >270 days, 5 were male and 5 were female.

### 3.3. Validation of cre-mediated exomap1 activation

**Fig. 2.**
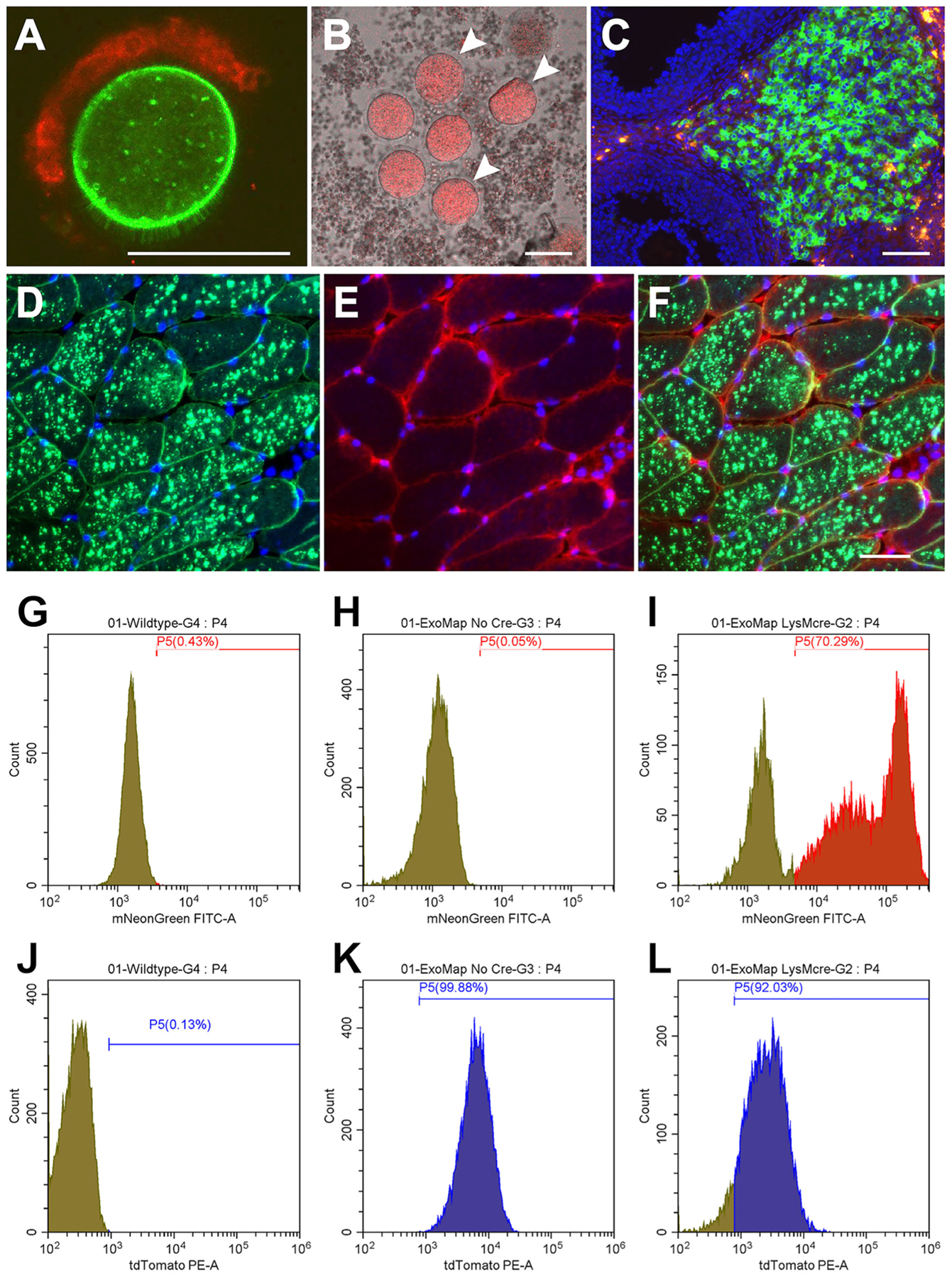
Cre-triggered expression of HsCD81mNG from the exomap1 transgene. (**A**) Fluorescence micrograph of an oocyte and a cluster of attached cumulus cells from an *exomap1*:*Zp3*-Cre mouse. Bar, 100 μm (**B**) Fluorescence micrograph of dispersed oocytes and granulosa cells from mechanically-disrupted small follicles of *exomap1*: *Cyp19a1*-Cre mice showing MTS-tdTomato expression in oocytes and only weak MTS-tdTomato expression in granulosa cells. White arrowheads point to oocytes. Bar, 100 μm. (**C**) Fluorescence micrograph of DAPI-stained section through an *exomap1*:*Cyp19a1*-Cre mouse ovary, showing strong *exomap1* transgene expression in cells of the corpus luteum, most of which expressed HsCD81mNG while some expressed MTS-tdTomato. At this exposure, the low level of MTS-tdTomato expression in DAPI-stained granulosa cells is not visible. Bar, 100 μm. (**D-F**) Fluorescence micrographs of a DAPI-stained cross section of quadriceps muscle from a tamoxifen-induced *exomap1*:*HSA*-MCM mouse, showing images of (D) HsCD81mNG fluorescence and DAPI, (E) MTS-tdTomato fluorescence and DAPI, and (F) HsCD81mNG, MTS-tdTomato, and DAPI. Bar, 100 μm. (**G-L**) Flow cytometry histograms (cell number vs fluorescence intensity) showing (G–I) green fluorescence and (J–L) red fluorescence of CD45^+^, CD11b + cells collected from the blood of (G, J) control mice, (H, K) *exomap1*^+/^ mice, and (I, L) *exomap1*:*LysM*-Cre mice.

To test whether the expression of Cre recombinase had its expected effects on the *exomap1* transgene, we crossed *exomap1*^+/^ carriers to a variety of Cre-expressing transgenic mouse lines. These experiments revealed that the *exomap1* mouse behaved largely as expected. For example, when we crossed *exomap1*^+/^ carriers with the oocyte-specific Cre driver *Zp3*-Cre mouse line,^53^ we found that *exomap1*:*Zp3*-Cre oocytes displayed strong expression of HsCD81mNG, while the somatic cumulus cells attached to the oocyte surface continued to express red MTS-tdTomato fluorescence (Fig. 2A). Furthermore, we found that *exomap1*:*Zp3*-Cre oocytes correctly localized HsCD81mNG to the plasma membrane, including the microvilli that protrude from the oocyte surface.^54^ As for the small amounts of HsCD81mNG fluorescence that we detected in internal structures of the oocyte, these likely reflect the presence of HsCD81mNG in deep invaginations of the plasma membrane invaginations^55–57^ and/or endolysosomal compartments (all plasma membrane proteins are internalized at some rate).

### 3.4. Variability in exomap1 transgene expression

The *exomap1* transgene is designed to express either MTS-tTomato or HsCD81mNG from the strong CAG promoter, with the hope and expectation that all cells in the body will express high levels of MTS-tdTomato and/or HsCD81mNG, depending on the timing of Cre expression. However, we observed a small number of circumstances in which we detected large variations in *exomap1* transgene expression. The most obvious example of this was in ovarian granulosa cells of *exomap1*:*Cyp19a1*-Cre female mice (generated by crossing *exomap1*^+/^ mice with an aromatase (*Cyp19a1*) driver line, *Cyp19a1*-Cre^42^). Aromatase is expressed in granulosa cells during follicular development, as well as after their terminal differentiation into luteal cells of the corpus luteum.^58–60^ Thus, when we examined ovaries from *exomap1*: *Cyp19a1*-Cre female mice treated with pregnant mare serum gonadotropin (PMSG), we expected to detect bright red fluorescence in granulosa cells of small immature follicles, with the percentage of HsCD81mNG-positive granulosa cells increasing as follicles matured, and peaking in luteal cells of the corpus lutem.^58–60^ However, when we mechanically disrupted small follicles to release their oocytes and granulosa cells, we observed bright red fluorescence in the oocytes but only weak red fluorescence in the granulosa cells (Fig. 2B). Furthermore, in ovarian tissue sections cut through small follicles, which would have just begun Cyp19a1 expression, we found that they had low levels of MTS-tTomato expression and no evidence HsCD81mNG expression (Fig. 2C), while the terminally differentiated luteal cells (i.e., luteinized granulosa cells) displayed robust expression from the *exomap1* transgene, most of which had recombined to express HsCD81mNG (though some expressed bright red MTS-tTomato fluorescence) (Fig. 2C). Furthermore, in tissue slices with large preovulatory follicles immediately prior to ovulation, we observed that many granulosa cells expressed HsCD81mNG. Taken together, these results show that *exomap1* transgene expression can be repressed in certain cell types, and that this repression can fade during differentiation/development. These results highlight the advantage of designing the *exomap1* transgene to express MTS-tdTomato, as this allows future users to test whether the *exomap1* transgene is expressed in their cell type(s) of interest by fluorescence microscopy.

### 3.5. Complex subcellular distribution of HsCD81mNG in skeletal muscle

Another variability that we encountered in our studies relates to the subcellular distribution of HsCD81mNG. As noted previously, CD81 is normally localized to the plasma membrane^5,6^ which we’ve recently found to be the primary site of exosome protein secretion in most cell types.^1,5,6,61^ As a result, expression of HsCD81mNG often leads to it’s accumulation at the plasma membrane (Fig. 2A). However, plasma membrane dynamics vary widely, and some cell types possess deep invaginations of the plasma membrane^55–57^. This is particularly true of muscle cells, which contain numerous transverse tubules. These structures are generated and remodeled by fission of tubular invaginations from the plasma membrane, as well as by fusion of endolysosomal tubules with the plasma membrane.^62^ Therefore, its not surprising that crossing *exomap1*^+/^ carriers with skeletal muscle Cre driver line *HSA--*Cre^ERT2^,^63^ and then activating Cre with tamoxifen, resulted in a complex pattern of HsCD81mNG fluorescence in DAPI-stained, adult quadriceps muscle tissue (Fig. 2D–F). Specifically, we detected strong HsCD81mNG fluorescence at the plasma membrane but also at numerous internal compartments. Interestingly, these images also highlighted the continued expression of MTS-tdTomato in the pericytes, fibroblasts, adipocytes, endothelial cells, and other support cells that surround each muscle fiber (Fig. 2E and F). We also performed additional control experiments that confirmed the expression of MTS-tdTomato and absence of HsCD81mNG expression in skeletal muscle of *exomap1*^+/^ mice, as well as the specific activation of HsCD81mNG expression in skeletal muscle of *exomap1*:*HSA*-Cre^ERT2^ mice (fig. S2).

### 3.6. Myeloid activation reveals the temporal lag between gain of HsCD81mNG and loss of MTS-tdTomato

Expression of Cre recombinase deletes the MTS-tdTomato ORF that separates the CAG promoter from the HsCD81mNG ORF. However, MTS-tdTomato protein and mRNA will always persist for some period of time after Cre-mediated deletion of the MTS-tdTomato ORF, and thus, we expect certain Cre driver experiments to generate cells that express HsCD81mNG yet still display red MTS-tdTomato fluorescence. The most obvious example of this was the persistence of MTS-tdTomato fluorescence in circulating myeloid (CD45^+^, CD11b^+^) cells of *exomap1*:*LysM*-Cre mice, which we generated by crossing *exomap1*^+/^ and *LysM*-Cre mice^64,65^ (LysM encodes monocyte lysozyme).

Flow cytometry of CD45^+^, CD11b^+^ blood cells from control and *exomap1*^+/^ carriers showed that they displayed only background levels of green fluorescence (Fig. 2G and H), whereas CD45^+^, CD11b^+^ blood cells from *exomap1*:*LysM*-Cre mice displayed bright green fluorescence in ~70% of cells (Fig. 2I). However, activation of HsCD81mNG expression in CD45^+^, CD11b^+^ blood cells from *exomap1*:*LysM*-Cre mice did not result in the loss of MTS-tdTomato fluorescence, as >90% of circulating monocytes from *exomap1*:*LysM*-Cre mice retained red fluorescence levels that were well above the background, and only ~3-fold lower than the level of red fluorescence of cells from *exomap1*^+/^ carrier mice (Fig. 2J–L).

These results highlight yet another advantage of incorporating the MTS-tdTomato ORF into the *exomap1* transgene, namely its ability to qualitatively report on the relative timing between *exomap1* transgene recombination and the moment of analysis. In this particular case, the high level of MTS-tdTomato fluorescence in CD45^+^, CD11b^+^ blood cells is consistent with the relatively short circulating half-life of these cells (they leave the blood after 1–2 days in circulation^66^) and the expression of *LysM*-Cre at or just prior to their entry into the blood. As for why HsCD81mNG expression was not detected in ~30% of CD45^+^, CD11b^+^ blood cells from *exomap1*:*LysM*-Cre mice, this may reflect the incomplete penetrance of the *LysM*-Cre driver, which is common to Cre driver lines.^65^

#### 3.6.1. Myeloid expression of HsCD81mNG releases exosome-associated mNeonGreen fluorescence into the blood

The expression of HsCD81mNG in circulating myeloid cells allowed us to test whether it was secreted from the cell in exosome-sized vesicles. To answer this question, we separated raw plasma from *exomap1*:*LysM*-Cre mice by size exclusion chromatography, followed by measurement of protein and of mNeonGreen fluorescence in each of the resulting fractions (excitation maximum 504 nm, emission maximum 517). These experiments revealed that the specific fluorescence of mNeonGreen was highest in the exosome-containing fractions that follow the void volume (Fig. S1).

### 3.7. Hepatocytes contribute ~15% of the blood exosome population

**Fig. 3.**
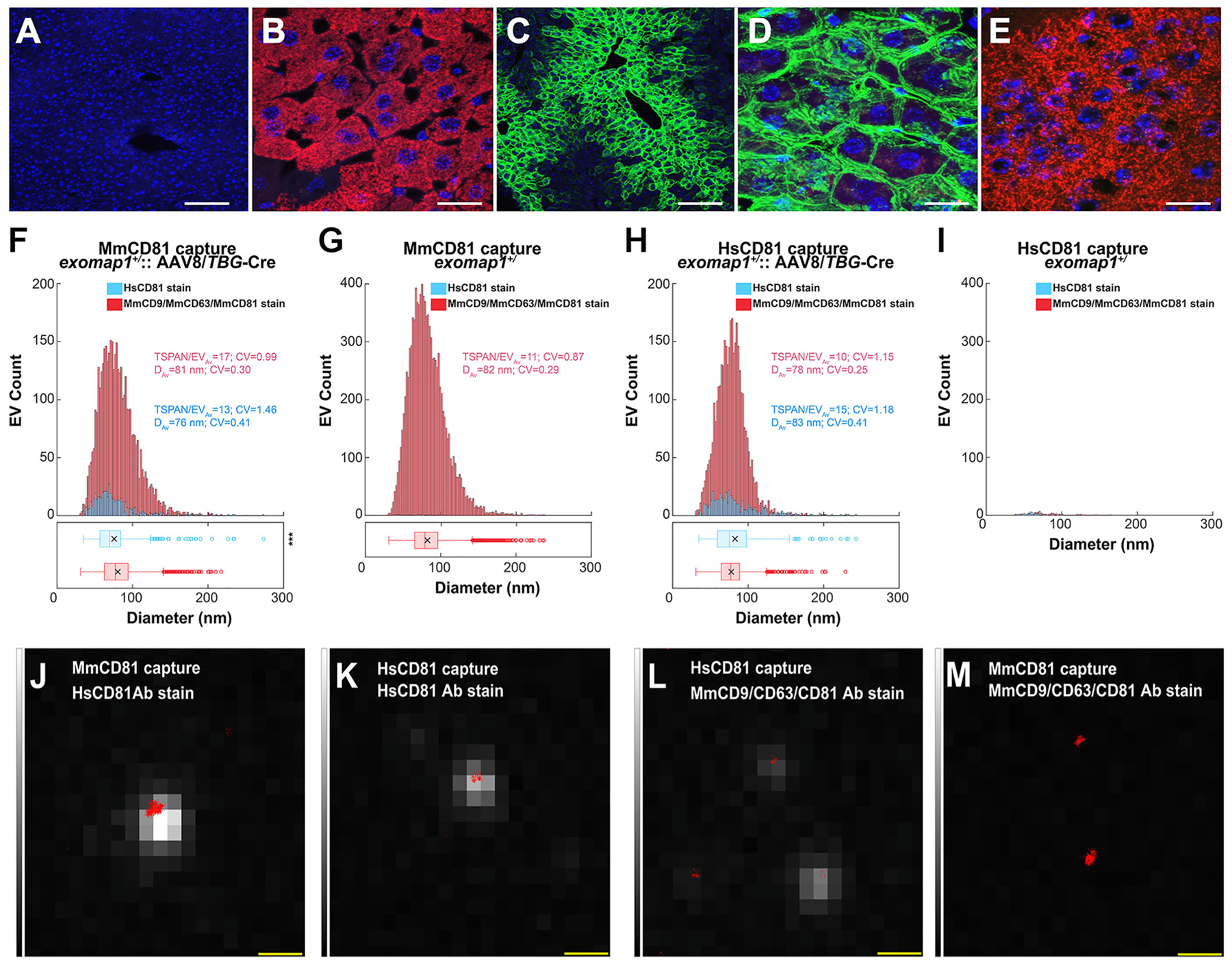
Hepatocytes cells load HsCD81mNG into mouse exosomes. (**A, B**) Fluorescence micrographs of liver sections from (A) a non-transgenic control mouse and (B) an *exomap1*^+/−^ mouse, stained with DAPI and imaged for (blue) DAPI and for (red) MTS-tdTomato fluorescence. Bar, 100 μm in A and 20 μm in B. (**C**) Fluorescence micrograph of a liver section obtained from an *exomap1*^+/^ mouse infected i. v. with AAV8/*TBG*-Cre virus, stained with DAPI, and imaged for (blue) DAPI and (green) HsCD81mNG. Bar, 100 μm. (**D**) Fluorescence micrograph of a liver section obtained from an *exomap1*^+/^ mouse infected i. v. with AAV8/*TBG*-Cre virus, stained with DAPI, and imaged for (blue) DAPI, (green) HsCD81mNG fluorescence, and (red) MTS-tdTomato fluorescence. Bar, 20 μm. (**E**) Fluorescence micrograph of a brain section obtained from an *exomap1*^+/^ mouse infected i. v. with AAV8/*TBG*-Cre virus, stained with DAPI, and imaged for (blue) DAPI and (green) HsCD81mNG fluorescence. Bar, 20 nm. (**F–I**) Histograms of qSMLM data collected for plasma-derived exosomes immunopurified on coverslips derivatized with antibodies specific for (F, G) MmCD81 or (H, I) HsCD81, from (F, H) an *exomap1*^+/^ mouse infected i. v. with AAV8/TBG-Cre virus or (G, I) an *exomap1*^+/^ mouse. Exosomes were stained with either (red) AF647-labeled anti-mouse tetraspanins (CD9, CD63, and CD81) or (blue) AF647-labeled *anti*-HsCD81. TSPAN/EV_Av_ denotes the average number of detected target proteins per vesicle, CV denotes the coefficient of variation, and D_Av_ denotes the mean diameter. The box and whisker plots below the histograms denote the mean (x), median (line), interquartile range (box), and and data points beyond 1.5-times the interquartile range (hollow dots). P values are included elsewhere (Table S1). (**J-M**) Fluorescence micrographs of individual exosomes interrogated by TIRF and by qSMLM, showing (greyscale) mNG fluorescence and (red dots) positions of vesicle-surface tetraspanins. These exosomes were collected from the plasma of an AAV8/*TBG*-Cre-infected *exomap1*^+/−^ mouse by immunoaffinity purification on (J, M) *anti*-MmCD81 or (K, L) *anti*-HsCD81 antibodies and stained with (J, K) AF647-labeled *anti*-HsCD81 antibody or (L, M) AF647-labeled antibodies to mouse exosomal tetraspanins. Bar, 500 nm.

Unlike myeloid cells, which release their exosomes directly into the blood, cells of solid tissues release their exosomes into interstitial fluids that are separated from the blood by endothelial barriers of varying porosity. Among the most porous is the sinusoidal endothelium of liver, as liver endothelial cells form large transcellular pores of ~50–300 nm diameter that allow free diffusion of exosomes from liver interstitial fluid into the blood. To determine whether HsCD81mNG expression in liver leads to the release of HsCD81mNG-positive exosomes into the blood, we injected *exomap1*^+/^ mice with a single dose (~5 × 10^10 particles) of a non-replicating adeno-associated virus (AAV) serotype 8 transfer vector (AAV8) that encodes a single gene consisting of thyroxine binding globulin (TBG) promoter upstream of the Cre recombinase ORF^67–71^. Control and transduced animals were maintained for 2–4 weeks to ensure sufficient time for HsCD81mNG to be expressed, and for residual AAV vector particles to be cleared from the body (AAV vectors have a half-life of at most 12 h^72^). Fluorescence microscopy of liver sections revealed that liver cells of normal control mice lacked red or green fluorescence (Fig. 3A) whereas hepatocytes of *exomap1*^+/^ mice expressed bright red fluorescence (Fig. 3B). In contrast, most hepatocytes of *exomap1*:AAV8/*TBG*-Cre mice expressed bright HsCD81mNG fluorescence, especially sinusoid-proximal hepatocytes (Fig. 3C). Furthermore, higher magnification images revealed that hepatocytes localized HsCD81mNG primarily to the plasma membrane, and that residual MTS-tdTomato fluorescence could still be detected in some HsCD81mNG-expressing cells (Fig. 3D). Transduction with AAV8/*TBG*-Cre did not, however, activate HsCD81mNG expression in brain, as brain tissue slices showed strong expression of MTS-tdTomato but no expression of HsCD81mNG (Fig. 3E).

To determine whether hepatocyte-derived, HsCD81mNG-containing exosomes made their way into the blood, plasmas were collected from *exomap1*^+/^ carriers and *exomap1*:AAV8/*TBG*-Cre mice, followed by immunopurification of exosomes on glass coverslips derivatized with monoclonal antibodies specific for either mouse CD81 (MmCD81) or HsCD81. The immunocaptured exosomes were then probed with Alexa Fluor 647 (AF647) conjugates of either a monoclonal antibody specific for HsCD81, or a cocktail of AF647-labeled monoclonal antibodies specific for MmCD81, MmCD9, and MmCD63.

After washing and fixation, these exosomes were interrogated by quantitative single-molecule localization microscopy (qSMLM).^44,46,73^ The cocktail of anti-mouse tetraspanin antibodies detected thousands of individual MmCD81-immunocaptured exosomes from both *exomap1*^+/^ and *exomap1*:AAV8/*TBG*-Cre mice (Fig. 3F and G; **red bars**). Normalization of these data by region-of-interest (ROI) and by volume (μL) of plasma assayed (Table 1) revealed that the numbers of exosomes per μL fell within an ~3-fold range (202/ROI/μL for *exomap1*:AAV8/*TBG*-Cre vs 517/ROI/μL for *exomap1*^+/^) (Table 1). The properties of exosomes in these two plasma samples were highly similar, as both had a mean diameter of ~80 nm, and an average of ~10–20 detected tetraspanins per exosome (a minimum of three was required for EV detection). However, when the plasma exosomes from the same *exomap1*: AAV8/*TBG*-Cre mouse were probed with the AF647-labeled *anti*-HsCD81 antibody (Fig. 3F; **blue bars**), we detected only 23 exosomes/ROI/μL, ~10 %of the number of exosomes detected using the anti-mouse antibodies to CD81, CD9, and CD63, which was 202/ROI/μL (Table 1). Based on these numbers, the percentage of plasma exosomes derived from liver hepatocytes appears to be ~11%.

These data also demonstrated that HsCD81mNG was loaded into bona fide mouse exosomes, as HsCD81-positive exosomes had the expected size (mean diameter of 76 nm), topology (outside out), and content (presence of MmCD81) of exosomes (Fig. 3F). Additional controls revealed that the background staining in this assay, determined using plasma from the *exomap1*^+/−^ mouse, was 0.7 exosomes per ROI/μL using anti-human CD81 antibody, compared to a total exosome detection rate of 517/ROI/μL using the anti-mouse tetraspanin antibody cocktail (Table 1; Fig. 3G; **blue bars**).

We next tested if plasma exosomes immunopurified on the *anti*-HsCD81 monoclonal antibody yielded paralogous results. We therefore subjected plasmas from the *exomap1*:AAV8/*TBG*-Cre and *exomap1*^+/^ mice to immunoaffinity purification of exosomes using glass coverslips derivatized with *anti*-HsCD81 antibody, then probed them with either a cocktail of AF647-labeled monoclonal antibodies specific for MmCD81, MmCD9, and MmCD63, or with AF647-labeled *anti*-HsCD81 antibody, then interrogated them by qSMLM. Significant numbers of hepatocyte-derived plasma exosomes from the *exomap1*:AAV8/*TBG*-Cre mouse were captured by the *anti*-HsCD81 antibody and stained positively the cocktail of antibodies to mouse tetraspanins (Fig. 3H, **red bars**). Moreover, these HsCD81mNG-containing exosomes had the size (mean diameter, 76 nm), topology (outside out), and content (mouse exosomal tetraspanins) of exosomes, showing once again that mouse cells loaded HsCD81 into bona fide exosomes carrying mouse exosome marker proteins.

As for the relative proportion of plasma exosomes that were immunocaptured on *anti*-HsCD81 antibodies, this appeared to be ~17%, as we detected 35 exosomes per ROI/μL on the *anti*-HsCD81-derivatized coverslips, compared to the 202 exosomes per ROI/μL that we detected on the MmCD81 immunocapture coverslips (Table 1). This yields an estimate of 17% for the hepatocyte contribution to plasma exosome populations, somewhat higher than the 11% estimate we calculated from the *anti*-HsCD81 immunostaining experiments described above (Fig. 3F and G). It is also consistent with the technical differences between these two experiments, as a single HsCD81mNG molecule can lead to immunoaffinity capture of a vesicle, while a minimum of three HsCD81mNG molecules need to be detected by immunostaining before we count it as a vesicle. This difference in sensitivity between immunocapture and immunodetection also explains why the number of exosomes immunopurified on the *anti*-HsCD81 capture antibodies and detected by the AF647-labeled *anti*-HCD81 antibody was 5 per ROI/μL, roughly 7-fold fewer than the 35 exosomes per ROI/μL we detected with the antibodies to mouse exosomal tetraspanins (Fig. 3H; Table 1). In addition, these data show that the Exomap1 mouse model produces a very small population of exosomes with four or more detected hCD81mNG, lending greater credence to the idea that it works like a tracer of exosome biology rather than a modulator of exosome content or function.

The same glass coverslips used in Fig. 3F and H were also examined by TIRF microscopy to detect mNeonGreen fluorescence. Consistent with the preceding considerations, we found that bright green HsCD81mNG fluorescence was most likely to be observed for exosomes that stained strongly with AF647-labeled *anti*-HsCD81 antibodies, regardless of whether they were captured on antibodies specific for MmCD81 or HsCD81 (Fig. 3J and K). As for those exosomes detected by staining with the cocktail of anti-mouse tetraspanin antibodies, green HsCD81mNG fluorescence was detected in some but not all of the exosomes captured with the *anti*-HsCD81 antibody (Fig. 3L). However, it was absent from most of the exosomes captured by the *anti*-MmCD81 antibodies (Fig. 3M), consistent with the fact that ~80–90% of these lack HsCD81mNG.

In addition to qSMLM and TIRF imaging studies, we also interrogated plasma exosomes from *exomap1*:AAV8/*TBG*-Cre mice by the coupled techniques of single-particle interferometric reflectance (SPIR) and immunofluorescence microscopy (IFM). These experiments revealed that some of the exosomes captured on *anti*-MmCD81 antibodies displayed bright green HsCD81mNG fluorescence, and that some of the exosomes captured on *anti*-HsCD81 antibodies stained strongly with antibodies specific for MmCD81 (Fig. S3), consistent with the results of our qSMLM experiments.

### 3.8. Neurons contribute only ~1% of exosomes present in plasma and cerebrospinal fluid

**Fig. 4.**
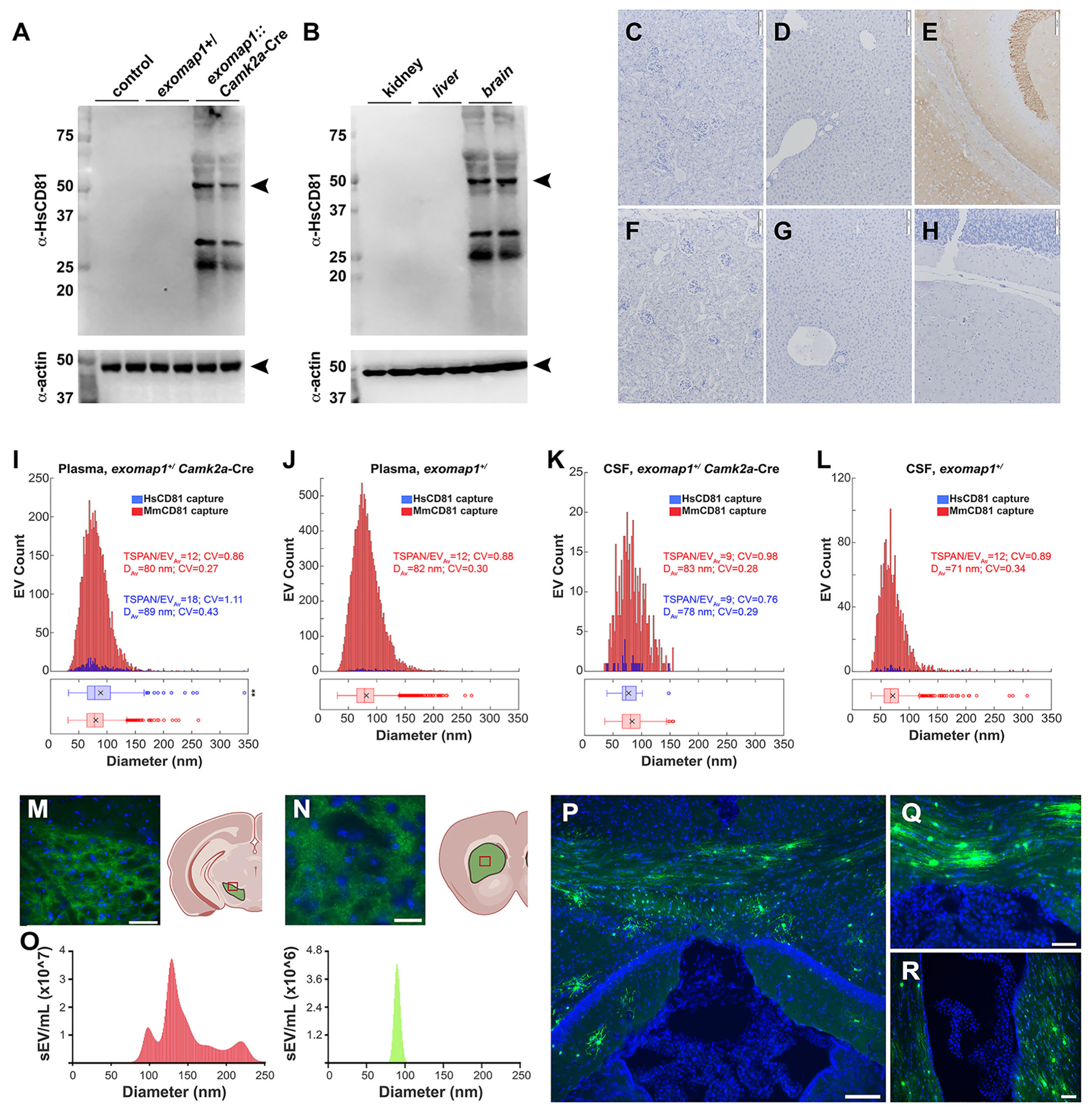
Neurons contribute ~1% of plasma exosomes. (**A**) Immunoblots of brain protein extracts from control mice, *exomap1*^+/−^ mice, *exomap1*:*Camk2a*-Cre mice probed with antibodies specific for (upper panel) HsCD81 and (lower panel) actin. MW size standards are in kDa. (**B**) Immunoblots of kidney, liver, and brain protein extracts *exomap1*:*Camk2a*-Cre mice probed with antibodies specific for (upper panel) HsCD81 and (lower panel) actin. MW size standards are in kDa. (**C–H**) Light micrographs of *anti*-HsCD81-stained tissues from *exomap1*:*Camk2a*-Cre mice and *exomap1*^+/^ mice, including (C, F) kidney, (D, G) liver, and (E, H) brain. Bar, 100 μm. (**I, J**) Histograms of single exosome immunophenotyping data collected by qSMLM, for raw plasma from (I) *exomap1*:*Camk2a*-Cre mouse and (J) *exomap1*^+/^ mouse. Exosomes were immunopurified on coverslips functionalized with (red bars) *anti*-MmCD81 antibody or (blue bars) *anti*-HsCD81 antibody, then stained with a cocktail of AF647-labeled antibodies to exosomal tetraspanins. TSPAN/EV_Av_ denotes the average number of detected target markers per vesicle, CV denotes the coefficient of variation, and D_Av_ denotes the mean diameter. The box and whisker plot below the histograms denote the mean (x), median (line), interquartile range (box), and point beyond 1.5-times the interquartile range (hollow dots). (**K, L**) Histograms of qSMLM data for raw CSF samples collected from (K) *exomap1*:*Camk2a*-Cre mouse and (L) *exomap1*^+/−^ mouse, immunopurified on coverslips functionalized with (red bars) *anti*-MmCD81 antibody or (blue bars) *anti*-HsCD81 antibody, then stained with a cocktail of AF647-labeled antibodies to exosomal tetraspanins. TSPAN/EV_Av_ denotes the average number of detected target markers per vesicle, CV denotes the coefficient of variation, and D_Av_ denotes the mean diameter. The box and whisker plot below histograms denote the mean (x), median (line), interquartile range (box), and point beyond 1.5-times the interquartile range (hollow dots). P values are included elsewhere (Table S1). (**M, N**) Fluorescence micrographs of DAPI-stained brain sections of *exomap1*:*Dat*-Cre mice showing HsCD81mNG fluorescence in (M) cells of the ventral tegmental area and (N) the axons in the striatum. Bar in A, 50 μm; bar in B, 25 μm. Cartoons to the right of each micrograph depict the brain location of these sections, and were generated in BioRender. (**O**) NTA histograms of raw CSF samples that had been passed through a 200 nm pore diameter size filter, showing (left histogram) the size distribution profile of all CSF sEVs and (right histogram) the size distribution profile of mNeonGreen-positive sEVs. (**P–R**) Blue/green fluorescence micrographs of DAPI-stained brain sections of *exomap1*^+/−^ mice injected with AAV5/*Rpe65*-Cre virus, showing HsCD81mNG fluorescence in cells of (**P**) the corpus callosum and hippocampus, (**Q**) posterior commissure, and (**R**) lateral ventricle. Bar in A, 100 μm; Bar in B and C, 50 μm.

In contrast to the high porosity of the liver endothelium, neurons are separated by blood brain and blood neuron barriers, which are impermeable to molecules larger than ~1 nm.^74^ To determine whether these barriers restrict the flow of neuronal exosomes into the blood, we first crossed *exomap1*^+/^ mice with a calcium/calmodulin-dependent protein kinase 2A (*Camk2a*)-Cre driver, which should induce the expression of HsCD81mNG in diverse populations of neurons.^75,76^ Immunoblot analysis of brain tissue extracts from control, *exomap1*^+/^ carrier mice, and *exomap1*:*Camk2a*-Cre mice showed that HsCD81mNG was strongly expressed in the *exomap1*:*Camk2a*-Cre brain samples (Fig. 4A) and not in their kidney or liver (Fig. 4B). Similar results were obtained when these tissues were processed for immunohistochemistry using *anti*-HsCD81 antibodies (Fig. 4C–H).

To determine whether the expression of HsCD81mNG in brain leads to the release of HsCD81mNG-positive exosomes into the blood, plasmas were collected from *exomap1*:*Camk2a*-Cre mice and *exomap1*^+/^ carriers, subjected to immunoaffinity purification of exosomes on glass coverslips derivatized with antibodies specific for either MmCD81 or HsCD81, and stained with a cocktail of AF647-labeled antibodies specific for MmCD9, MmCD63, MmCD81, and HsCD81. Washed and fixed samples were then examined by qSMLM.

Thousands of EVs with the expected size (~80 nm diameter), topology (outside out), and content (mouse exosomal tetraspanins) of exosomes were detected in plasma samples from both *exomap1*:*Camk2a*-Cre mice and *exomap1*^+/^ mice (Fig. 4I, J; **red bars**). Furthermore, normalization to ROI and amount of plasma assayed indicated a concentration of 259 detected exosomes per ROI/μL for the *exomap1*: *Camk2a*-Cre plasma sample and 648 detected exosomes per ROI/μL for the *exomap1*^+/^ plasma sample (Table 2). In contrast, the concentration of *exomap1*:*Camk2a*-Cre plasma exosomes captured on the *anti*-HsCD81 coverslip was far lower, only 3.3 per ROI/μL (Table 2), indicating that neuron-derived exosomes make up only ~1% of the plasma exosome population (3.3/259), against a background staining rate of ~0.2% in plasma from the *exomap1*^+/^ [(1.7/ROI/μL)/(648/ROI/μL)] (Table 2).

The creation of *exomap1*:*Camk2a*-Cre mice also allowed us to test the neuronal contributions to cerebrospinal fluid (CSF). Raw CSF was collected from *exomap1*:*Camk2a*-Cre and *exomap1*^+/^ carriers, followed by immunopurification of CSF exosomes on glass coverslips derivatized with antibodies specific for either MmCD81 or HsCD81 for *exomap1*: *Camk2a*-Cre (Fig. 4K) or *exomap1*^+/^ (Fig. 4L). Bound exosomes were then stained with the cocktail of AF647-labeled antibodies specific for MmCD81, MmCD9, MmCD63, and HsCD81 and examined by qSMLM. Exosomes captured from CSF of *exomap1*:*Camk2a*-Cre and *exomap1*^+/^ carriers had similar sizes (~70–80 nm mean diameter) and tetraspanin densities (~9–12 per exosome) as plasma exosomes, regardless of whether they were captured on *anti*-MmCD81 or *anti*-HsCD81 antibodies (Fig. 4K, L). However, the concentration of exosomes detected on the *anti*-MmCD81 coverslip was 42 per ROI/μL, whereas we detected 0.4 exosomes per ROI/μL on the *anti*-HsCD81 coverslip (Table 3). These results indicate that central nervous system (CNS) neurons contributed only ~1% to the CSF exosome population. The background in this assay was ~0.4% for CSF from *exomap1*^+/^ carriers [(0.7/ROI/μL)/(161/ROI/μL)] (Table 3), raising the possibility that the neuronal contribution to CSF exosome population might be even lower than 1%. Taken together, these results indicate that tight endothelial and epithelial barriers may block the flow of neuron-derived exosomes into the CSF.

### 3.9. Activation of HsCD81mNG expression in the midbrain

Midbrain neurons, including those that express the dopamine transporter (*Dat*), are located proximal to the central aqueduct and may therefore be responsible for much of the neuronal exosome contribution to the CSF. To explore this possibility, we crossed *exomap1*^+/^ mice with a dopamine transporter gene Cre driver, *Dat*-Cre.^77^ Fluorescence microscopy of brain sections from *exomap1*:*Dat*-Cre mice showed HsCD81mNG was expressed in neurons of the ventral tegmental area (Fig. 4M) and axons in the striatum (Fig. 4N). To determine the percentage of *exomap1*:*Dat*-Cre CSF EVs that contained HsCD81mNG, we collected CSF, passed it through a 200 nm pore diameter filter, and examined the filtered CSF by nanoparticle tracking analysis (NTA) coupled to fluorescent particle detection. These experiments revealed that mouse CSF contains a complex population of sEVs (Fig. 4O, left panel), that the HsCD81mNG-containing exosomes had a mean diameter of ~80 nm (Fig. 4O, right panel), but only ~1% of the CSF sEVs displayed HsCD81mNG fluorescence.

### 3.10. Exomap1 activation in adult brain

In our final experiments, we tested whether HsCD81mNG expression could be induced in diverse brain cells of adult mice by subjecting *exomap1*^+/^ carrier mice to intracerebroventricular (ICV) injection with AAV5/*Rpe65*-Cre, which drives the expression of Cre in a wide array of cell types (astrocytes, glia, microglia, neurons, etc.). Three weeks after injection, brain tissue slices were stained with DAPI and examined by fluorescence microscopy. The resulting images revealed the presence of HsCD81mNG fluorescence in individual cells in white matter tracks, including the corpus callosum (Fig. 4P), the posterior commissure (Fig. 4Q), and in subventricular regions surrounding the lateral ventricle (Fig. 4R). In addition to demonstrating that the *exomap1* transgene could be activated in adult brain cells, these results highlight yet another advantage of viral Cre delivery, namely that viral delivery can be adjusted to activate the HsCD81mNG transgene in only one or a few cells, which may prove useful in future studies aimed at mapping cell-to-cell exosome traffic within the brain and other solid tissues.

## 4. Discussion

The data presented in this paper validate the design of the *exomap1* transgene and Exomap1 mouse, demonstrate the scope of its operational utility, and show that it can be used to determine the cell type-specific contributions to biofluid exosome populations, data that’s critical to the development of exosome-based liquid biopsies of human health and disease.

## 5. Validation of exomap1 transgene design principles

The operational utility of the *exomap1* transgene and Exomap1 mouse results from their unique design, which was guided by an array of prior observations. Some of these observations relate to the properties of HsCD81mNG, including (***i***) the 25 years of data that shows CD81 to be the most highly-enriched exosome cargo protein yet described^1,3,5,6,32,78^; (***ii***) the lack of enrichment of CD81 in the larger, microvesicle class of EVs^32^; (***iii***) the fact that CD81 is an integral membrane tetraspanin and therefore never released from cells as free protein; (***iv***) the commercial availability of monoclonal antibodies that are specific for extracellular epitopes of HsCD81 and MmCD81; (***v***) the fact that mNeonGreen is ~2.5-fold brighter than GFP(78); (***vi***) our prior observation that HsCD81mNG is secreted from cells in exosomes^79^; (***vii***) the fact that CD81 protein fragments are not shed from the surface of cells or exosomes,^5^ and (***viii***) the fact that using an exosome marker other than CD63 avoids the dominant negative effects of high-level CD63 expression, which include neonatal lethality in rats,^80^ inhibition of endocytosis,^61^ and the artefactual loading of lysosomal proteins into exosomes.^61,81^

Other considerations that informed the design of the *exomap1* transgene and mouse were (***ix***) the consistency of expression that comes from transgene insertion at the H11 safe harbor locus of the genome^34^; (***x***) the extensive evidence that CAG-driven transgenes are expressed in most mouse cell types^82^; (***xi***) the exquisite control of HsCD81mNG expression afforded by Cre-lox system^34^; and (***xii***) the fact that an upstream ORF encoding MTS-tdTomato allows easy validation of *exomap1* transgene expression in the cell type of interest, while also reporting on the timing of Cre-mediated transgene activation.

The data presented here indicate that each of these design elements worked largely as expected. Insertion of the CAG-driven *exomap1* transgene in the H11 locus created an *exomap1* mouse that expressed MTS-tdTomato in the vast majority of mouse cells, showed no expression of HsCD81mNG in the absence of Cre recombinase, was inherited in a Mendelian fashion, and displayed no signs of adverse effects on animal health. In addition, the *exomap1* transgene responded appropriately to Cre recombinase by inducing HsCD81mNG, while also triggering the decline/loss of MTS-tdTomato fluorescence, which proceeds with an informative time lag. Moreover, we found that the *exomap1* transgene was activated by all eight Cre drivers tested, including two Cre-expressing AAV vectors.

However, the most important validation of the *exomap1* mouse was its faithful reproduction of CD81 biogenesis, which includes its trafficking to the plasma membrane, which appears to be the primary site of exosome biogenesis in most cell lines,^1,5,6,61^ as well as its efficient loading into secreted vesicles that had the correct size (~80 nm), topology (outside out), and composition (presence of mouse exosome markers CD81, CD9, and/or CD63) of exosomes. Importantly, our data also confirmed the prior demonstration that HsCD81mNG was not enriched in the large, microvesicle class of EVs.^32^ Our data also demonstrated that exosomal HsCD81mNG can be detected by immunological techniques, using antibodies specific for human CD81, and by the specific fluorescence of the mNeonGreen moiety that HsCD81mNG carries with it to the plasma membrane and into the lumen of exosomes. In fact, we found that nmost of the specific mNeonGreen fluorescence that HsCD81mNG-expressing cells released into the blood co-fractionated with exosomes.

### 5.1. Neuron-derived exosomes comprise ~1% of CD81-positive exosomes of plasma and CSF

The data presented here also demonstrated that the Exomap1 mouse can be used to map the cell type-specific contribution to biofluid exosome populations, and more importantly, revealed that neurons contribute only ~1% of the exosomes that are present in plasma, and also contribute only ~1% of the exosomes that are present in CSF. This observation has important implications for the exosome-based liquid biopsy of neuronal health and disease. The most obvious of these is that plasma may be just as enriched in neuron-derived exosomes as CSF, a fluid that is in short supply and only obtained by invasive, risky medical procedures.

The other major implication of our paper is that bulk analysis of plasma exosomes is unlikely to yield useful information on neuronal health and disease, whereas selective purification of neuron-derived exosomes from the plasma may yield useful information. Given that the Exomap1 mouse model was designed so that HsCD81mNG-containing exosomes can be selective immunocaptured means that this mouse model can be used to interrogate the immunophenotypes of neuron-derived exosomes, by capturing them from the plasma of *exomap1*:*Camk2a*-Cre mice wit anti-HsCD81 antibodies, then probing them with antibodies specific to neuron-expressed membrane proteins. While these studies are in their infancy, this approach may be more productive than prior studies, which have yielded more controversy than solid progress.^83–88^

### 5.2. Hepatocytes contribute ~15% of plasma exosomes

In addition to discovering that neurons contribute only ~1% of plasma exosomes, we found that hepatocytes contribute ~15% of plasma exosomes. Specifically, our sensitive single extracellular vesicle nanoscopy (SEVEN) assay yielded an estimate of ~17% (immunocapture with HsCD81) while a paralogous assay that requires higher HsCD81 signal levels (minimum of 3 detected proteins for immunostaining) yielded an estimate of 11%. We achieved these estimates using a relatively new exosome assay that employs direct immunocapture from raw biofluids and rigorous immunophenotyping by qSMLM^44–46^. As for the reliability of this experimental approach, the fact that our estimates of hepatocyte contributions are similar to the ~9–15% estimate arrived at by Li et al.^89^ using a different model and experimental approach (SPIR-IFM imaging) offers some independent validation of our model, experimental strategy, results, and interpretations.

Another validation of our approach is that our data and interpretations match the anatomy and physiology of the barriers that separate neurons and hepatocytes from the blood. The blood brain and blood neuron barriers are impervious to molecules larger than 1–5 nm in size,^90,91^ and as a result, there is no way for neuron-derived exosomes to diffuse passively from the interstitial fluids of the brain into the blood. Thus, our estimates of ~1% neuronal contribution to plasma and CSF exosome populations seems to match what’s already known about neuronal tissue permeabilities. In contrast to neurons, hepatocytes are separated from the blood by liver sinusoidal veins that are lined with endothelial cells that form large transcellular pores of ~50–300 nm diameter, more than large enough to allow hepatocyte-derived exosomes to diffuse from liver the interstitial fluid into the blood, consistent with themuch larger estimate of liver-to-blood exosome flow.

In short, our results indicate that cell type-specific contributions to plasma exosome populations will be determined primarily by the number of a particular cell type in the body, the porosity of the endothelial or epithelial barriers that separate them from the blood, and the extent to which they are transcytosed across these barrier cells. From this perspective, we anticipate that future studies of cell type-specific contributions of exosomes to plasma will show that most plasma exosomes are derived from blood, endothelial, liver, and muscle cells, as these make up the bulk of cells in the body and/or have direct contact with the blood.

### 5.3. Difference and advantages of the Exomap1 model

Taken together, the results presented here indicate that the *exomap1* mouse is a useful tool for *in vivo* studies of exosome biology. While this is not the first animal model of exosome biogenesis to be developed, we believe that that the Exomap1 mouse has advantages over other transgenic animal models that have developed for studying exosome biology.

These include features that are unique to the Exomap1 mouse, as it is the only mouse model that:

- is based on the most highly enriched exosome protein, CD81 (5,6,8,64);
- uses mNG, which is ~2.5-fold brighter than GFP(78);
- uses a cargo protein that is NOT enriched in other sEVs or the larger, microvesicle class of EVs^32^;
- employs an upstream visual marker, MTS-tdTomato, which (***i***) allows visualization of transgene expression in the cell type of interest, (***ii***) identifies non-expressing cell types, (***iii***) provides a timer of Cre activation, and (***iv***) reduces unwanted HsCD81mNG expression from leaky Cre drivers, as it placed the loxP sites >1 kb apart^92^; and
- loads only a small number of HsCD81mNG marker proteins per exosome (most have fewer than 4 detected HsCD81mNG molecules per vesicle).

Other advantages of the Exomap1 mouse are not unique but are still important:

- by avoiding the use of CD63, the Exomap1 mouse avoids the lethal effects of high-level CD63 expression^80^;
- by avoiding the use of CD63, the Exomap1 mouse avoids the dominant negative effects of CD63 on endocytosis and exosome content,^61^ which may confound results obtained using CD63-GFP models^80,89,93–95^;
- by using a human exosome marker protein, the *exomap1* mouse allows the immunocapture and immunodetection of marked exosomes, whereas models based on a mouse exosome marker protein^95^ do not;
- by using an intact exosomal marker protein, the *exomap1* mouse avoids potential artefacts that may arise when using a heavily mutated exosome marker protein^96^; and
- by inserting the *exomap1* transgene into a safe harbor locus, the *exomap1* mouse avoids position effect variations in transgene expression and activation that can arise from random integration of a transgene^80,93,94,97^;

Based on these various considerations, the mouse model most similar to the Exomap1 mouse is the TIGER mouse, as it uses CD9-GFP to avoid the lethality and dominant negative effects of CD63 overexpression, has an upstream lox-stop-lox cassette that confers Cre-triggered activation, and uses the strong CAG promoter to drive high-level expression in most cell types of interest.^97^ However, the TIGER mouse transgene has a few minor drawbacks, as it was inserted randomly in the genome, with unknown effects on cell type-specific transgene expression, lacks a visualizable upstream marker protein for the assessment of transgene expression in cell types of interest, and lacks the unique qualities of CD81..

### 5.4. Limitations of the Exomap1 model

The Exomap1 mouse is susceptible to the limitations shared by all Cre-lox-based mouse models, including incomplete penetrance of Cre-mediated transgene activation, and variable expression from the CAG promoter/H11 locus in different cell types and/or different stages of differentiation and development.^98^ We observed some evidence for these limitations in this study but they were in general rare and minor.

### 5.5. Data analysis and presentation

Statistical analysis involved calculation of averages and standard error of the mean, with pairwise differences evaluated for likelihood of null hypothesis using Student’s t-test, or ANOVA for experiments eval-uating more than 2 sample sets. P-values for exosome diameters and marker protein abundance/EV from qSMLM data were calculated after logarithmic transformation to eliminate the bias of distribution skew-ness. Histograms and scatter plots were generated using Excel and Matlab R2022a. Images were imported into Adobe Photoshop and figures were assembled in Adobe Illustrator. Image data was adjusted for brightness only.

## Supplementary Material

Supplementary Data

## Figures and Tables

**Table 1:** Numbers of exosomes detected in plasma samples from *exomap1* and *exomap1*::AAV8/*TBG-Cre* mice. Plasmas were collected from *exomap1*^+/^ and *exomap1*::AAV8/*TBG*-Cre mice followed by immunopurification of sEVs on coverslips derivatized with *anti*-MmCD81 or *anti*-HsCD81 monoclonal antibodies. The immunopurified sEVs were then stained using a cocktail of fluorescently-tagged antibodies specific for mouse CD9, CD63, and CD81 (MmTSPAN stain) or the *anti*-HsCD81 antibody only (HsCD81 stain). Data reflect the number of detected exosomes per region of interest (ROI), normalized to 1 μL of plasma. The experiments were performed as 20 technical replicates of two separate coverslips.

		Mean	Median	SEM	CV
***exomap1* ::AAV8/*TBG*-Cre MmCD81 capture/MmTSPAN stain**	EV/μL	202	198	5.8	0.13
***exomap1*^+/^ MmCD81 capture/MmTSPAN stain**	EV/μL	517	535	19.9	0.17
***exomap1* ::AAV8/*TBG*-Cre MmCD81 capture/HsCD81 stain**	EV/μL	23.0	22.0	1.4	0.28
***exomap1*^+/^ MmCD81 capture/HsCD81 stain**	EV/μL	0.7	0.0	0.2	1.16
***exomap1* ::AAV8/*TBG*-Cre HsCD81 capture/MmTSPAN stain**	EV/μL	35.4	32.4	2.6	0.33
***exomap1*^+/^ HsCD81 capture/MmTSPAN stain**	EV/μL	0.8	0.7	0.1	0.70
***exomap1* ::AAV8/*TBG*-Cre HsCD81 capture/HsCD81 stain**	EV/μL	4.9	5.1	0.4	0.33
***exomap1*^+/^ HsCD81 capture/HsCD81 stain**	EV/μL	0.5	0.3	0.1	1.13

**Table 2:** Numbers of exosomes detected in plasma samples from *exomap1* and *exomap1*::*camk2a*-Cre mice. Plasmas were collected from *exomap1*^+/^ and *exomap1*::*camk2a*-Cre mice, followed by immunopurification of sEVs on coverslips derivatized with *anti*-MmCD81 or *anti*-HsCD81 monoclonal antibodies. The immunopurified sEVs were then stained using a cocktail of fluorescently-tagged antibodies specific for mouse CD9, CD63, and CD81 and human CD81. Data are from 20 ROIs of two independently-imaged coverslips (values normalized to 1 μL of plasma).

		Mean	Median	SEM	CV
***exomap1*::*camk2a*-Cre MmCD81 capture**	EV/μL	259	254	7.1	0.12
***exomap1*::*camk2a*-Cre HsCD81 capture**	EV/μL	3.3	3.2	0.2	0.28
***exomap1*^+/^ MmCD81 capture**	EV/μL	648	654	29	0.20
***exomap1*^+/^ HsCD81 capture**	EV/μL	1.7	1.3	0.2	0.60

**Table 3:** Numbers of exosomes detected in CSF samples from *exomap1* and *exomap1*::*camk2a*-Cre mice. CSF was collected from *exomap1*^+/^ and *exomap1*:: *camk2a*-Cre mice followed by immunopurification of sEVs on coverslips derivatized with *anti*-MmCD81 or *anti*-HsCD81 monoclonal antibodies. The immunopurified sEVs were then stained using a cocktail of fluorescently-tagged antibodies specific for mouse CD9, CD63, and CD81 and human CD81. Data are from 10 ROIs (values normalized to 1 μL CSF).

		Mean	Median	SEM	CV
***exomap1::camk2a*-Cre MmCD81 capture**	EV/μL	42	43	3.1	0.24
***exomap1::camk2a*-Cre HsCD81 capture**	EV/μL	0.4	0.3	0.1	0.71
***exomap1*^+/^ MmCD81 capture**	EV/μL	161	156	15	0.29
***exomap1*^+/^ HsCD81 capture**	EV/μL	0.7	0.7	0.1	0.36

## Data Availability

*Exomap1* mice will distributed through a commercial provider.

## Abbreviations:

AAV: adeno-associated virus
BBB: blood brain barrier
BNB: blood neuron barrier
CNS: central nervous system
CSF: cerebrospinal fluid
EV: extracellular vesicle
HSA: human skeletal muscle actin
IB: immunoblot
ICV: intracerebroventricular
IFM: immunofluorescence microscopy
lEV: large EV
mNG: mNeonGreen
NTA: nanoparticle tracking analysis
ORF: open reading frame
qSMLM: quantitative single-molecule localization microscopy
sEV: small EV
SEVEN: single extracellular vesicle nanoscopy
SPIR: single-particle interferometric reflectance
TIRF: total internal reflection fluorescence
